# Effects of the illicit market on the price elasticity of cigarette consumption in Brazil

**DOI:** 10.1136/tc-2022-057787

**Published:** 2023-12-01

**Authors:** Jose Angelo Divino, Philipp Ehrl, Osvaldo Candido, Marcos Aurelio Pereira Valadao

**Affiliations:** 1 Catholic University of Brasilia, Brasilia, Brazil; 2 Getulio Vargas Foundation School of Public Policy and Government, Brasilia, Brazil

**Keywords:** Tobacco industry, Public policy, Taxation

## Abstract

**Background:**

An important element to consider in tobacco tax policy is the illicit market of cigarette sales. The objective of this paper is to provide estimates of both conditional and unconditional price elasticities of cigarette consumption in the licit and illicit markets in Brazil.

**Methodology:**

Microdata from the National Health Survey in 2013 and 2019 are used to estimate conditional and unconditional price elasticities of cigarette consumption in the licit and illicit cigarette markets by income quartiles and age cohorts. The identification is based on brand information and the official minimum cigarette price defined by the government, as sales below this price are prohibited and illegal.

**Findings:**

The results, robust to potential endogeneity, indicate that there is joint statistical difference in price elasticities across age cohorts and income groups by market type. However, individuals smoking illicit cigarettes, regardless of age cohort and income quartiles, are less sensitive to price changes than those consuming licit brands.

**Conclusions:**

The illicit cigarette market prevents the government from collecting tobacco tax revenues and weakens the social reach of price-oriented antismoking public policies. Fighting the illicit trade should be a major concern of public policies aiming at reducing cigarette consumption.

**WHAT IS ALREADY KNOWN ON THIS TOPIC**

- Price elasticity of cigarette consumption has been estimated for many countries around the world. Higher prices decrease consumption and the sensitivity of consumers to price changes depends on their level of addiction.

**WHAT THIS STUDY ADDS**

- First study to estimate price elasticity of cigarette consumption by age cohort and income quantiles in both illicit and licit markets.
- Smokers of illicit cigarettes in Brazil, regardless of age cohort and income quartiles, are less sensitive to price changes than those consuming licit brands.

**HOW THIS STUDY MIGHT AFFECT RESEARCH, PRACTICE OR POLICY**

- Fighting the illicit trade should be a major concern of public policies aiming at increasing tobacco tax collection and the social reach of price-oriented antismoking public policies.

## Introduction

The empirical literature on tobacco economics is extensive and includes applications to several countries around the world. Theoretically, it relies on the studies of addictive goods, as pioneered by Stigler and Becker,^1^ Becker and Murphy,^2^ among others. Chaloupka,^3^ for instance, applied the rationality model of Becker and Murphy.^2^ A key insight from a theoretical and empirical standpoint is that addictive goods, such as cigarettes, are less price elastic than non-addictive goods.

The price elasticity is an important tool for applied researchers and policy makers because it indicates how effective price-based tobacco control measures, such as tax increases, can be. Having price elasticities for specific groups in the population provides additional insights regarding the distribution of the overall tax burden and whether tax increases will be progressive or regressive. Regarding the estimates by age or income groups, the literature is much less consolidated (Ross and Chaloupka^4^). Another important aspect, particularly for low-income and middle-income countries, that is rarely addressed is how the presence of a large illicit cigarette market affects the smoker’s price sensitivity. Furthermore, few studies estimate price elasticities simultaneously for the licit and illicit markets as in the present case. Due to variation in geographical coverage, sampling criteria and methodological approaches, comparison among them in review studies tends to be more complex. See, for instance, Wilson *et al*.^5^

Brazil is usually referred to as a successful case of tobacco control policies, having experienced a persistent and substantial reduction in the prevalence of smoking since 2006. However, an important element to consider in tobacco tax policy is the illicit market of cigarette sales. Data from the Federal Revenue Service (Receita Federal do Brasil) indicate a decrease close to 50% in the licit production of manufactured cigarettes from 2006 to 2018. At the same time, the size of the illicit cigarette market in Brazil fluctuates, depending on the period and method of estimation. Thus, to some extent, Brazil could be experiencing a substitution of licit cigarettes by illicit ones, which would require additional policy measures focused on curbing illicit trade.

The objective of this study is to estimate both conditional and unconditional own price elasticities of cigarette consumption in the whole market as well as in the licit and illicit markets of cigarette sales in Brazil. Based on the National Health Survey (PNS) from 2013 and 2019 (Brazilian Institute of Geography and Statistics, IBGE^6^), we estimate price elasticities of cigarette consumption by income quartile and age cohorts. We apply a novel way to identify the illicit market based on cigarette purchases below the official floor price defined by the government and brand classification as licit or illicit by the Brazilian Health Regulatory Agency. The potential endogeneity problem is accounted for by using the state average prices instead of individually reported prices in the regressions. Ross and Chaloupka^4^ and Sweis and Chaloupka^7^ used the same approach and argued that a geographically based measure of individual prices is an effective way to avoid the endogeneity bias in models of cigarette demand.

In line with McKelvey,^8^ we argue that the proposed correction in Deaton^9^ is unlikely to improve the results and the required assumptions are not valid in the current setting. Deaton’s correction was developed for household survey data, whereas we are using individual survey with a different questionnaire structure. First, under the plausible assumptions that smokers did not buy different brands in their last purchase and that it is representative for their usual consumption behaviour, the inferred unit price for cigarettes is more precise than those inferred in household consumption surveys. Our procedure is an adaptation of Deaton^9^ for individual-level prices. By doing so, we reduce the risk of reverse causality in the estimated models. The estimated elasticities suggest that individuals purchasing cigarettes in the illicit market are less sensitive to price variations than individuals purchasing in the licit market. Our results also complement the literature which has produced mixed results regarding the variation of price elasticities among age and income groups (Ross and Chaloupka^4^). Wasserman *et al* 10 and Chaloupka^3^ find that young adults are less price sensitive compared with older individuals. Choi^11^ observes that lower income smokers in Korea are more responsive to changes in cigarette prices. However, employing different methods for other countries has produced opposing results; see the reviews by Wilson *et al*,^5^ Levy *et al* 12 and Bafunno *et al*.^13^

Previous studies estimated price elasticities of cigarette consumption for Brazil, under different set-ups, include Carvalho and Lobão,^14^ Iglesias,^15^ Ribeiro and Pinto^16^ and Lampreia *et al*.^17^ However, none of them explored the effects of the illicit market on the estimates.

## Material and methods

To describe the smoking behaviour of the Brazilian population and to estimate the sensitivity of cigarette consumption to price changes, we used two individual-level surveys: the PNS from 2013 and 2019 (IBGE^6^). Both surveys are representative of the population and conducted by IBGE. Their broad questionnaire is highly comparable and includes a special section on smoking behaviour. The PNS has a household and an individual component. We focused on the latter questionnaire because smoking behaviour is essentially an individual choice. Using the provided sample weights, the derived statistics are representative for the entire Brazilian population. Further details on these surveys are available in Szwarcwald *et al*.^18^

The survey respondents indicate how many cigarettes they smoke per day and at what age they started smoking. A key distinction of the PNS data in comparison to commonly used household survey price data is that the information regarding the quantity and total price of cigarettes refers to the last individual purchase. The interviewee reports the unit of purchase (sticks, packs or cartons) and the total price paid. This information is then processed by the authors to calculate the implied price of a 20-cigarette pack. The first advantage of this type of question is that it includes all kinds of markets where cigarettes are sold. Consequently, it will be fruitful to exploit the official minimum price for a 20-cigarette pack, which in 2013 was equal to BRL3.50 and by 2019 has been raised to BRL5.00. Purchases below this floor price shall have occurred in the illicit market because it is forbidden by the law to sell cigarettes below this price in the legal market. Prices and changes in prices must be reported to the Federal Revenue Service and any change in the retail price must also be informed because ad valorem taxes are based on this price. In addition, cigarette packs are subject to tax stamps as a control system. No promotional sales are allowed, and no cigarette can be legally sold at a price below the official floor price. A remaining concern is that cigarettes may still be illicit and sold above the minimum price. For the 2019 PNS data, we can additionally use information about the cigarette brands that allowed the IBGE to classify them as licit or illicit. Overall, in both PNS data sets, about one-third of all consumers purchased cigarettes below the official price floor. For better comparability of the two data sets, monetary variables were deflated by the wide consumer price index (IPCA (Índice Nacional de Preços ao Consumidor Amplo)) calculated and released by the IBGE. Table 1 provides a brief characterisation of the illicit market in 2013 and 2019 in terms of size and the share of cigarettes sold below the minimum price. For a more complete description, see online supplemental appendix table A.1.

10.1136/tc-2022-057787.supp1 Supplementary data

**Table 1:** Characterisation of the illicit cigarette market according to the PNS surveys

Year	Size in terms of cigarette consumption (%)	Size in terms of total number of smokers (%)	Share of cigarette purchases below the minimum price (%)
2013	35.8	31.3	100
2019	40.6	35.4	90.6

In the PNS 2013, the definition of illicit market is based on cigarettes sold below the official minimum price, while in the PNS 2019 the definition is based on the reported purchased brand and corresponding classification as licit or illicit by the Brazilian Health Regulatory Agency (Anvisa). The latter is more accurate because some illicit cigarettes are sold above the minimum price, as illustrated in the fourth column.

PNS, National Health Survey.

The price elasticity of cigarette consumption measures the per cent change in cigarette consumption due to a per cent change in the cigarette price. To account for the potentially different effects of a tobacco price increase over the income and age distributions, price elasticities are estimated by income quartiles and age cohorts. We estimate these equations jointly for the full sample of smokers, which includes 2 years and two sources of cigarette purchase, either the licit or illicit market. As an extension, we include an indicator variable for the legal market and interact it with all explanatory variables, so that price elasticities in both markets are identified from a single estimation.

**Figure 1:**
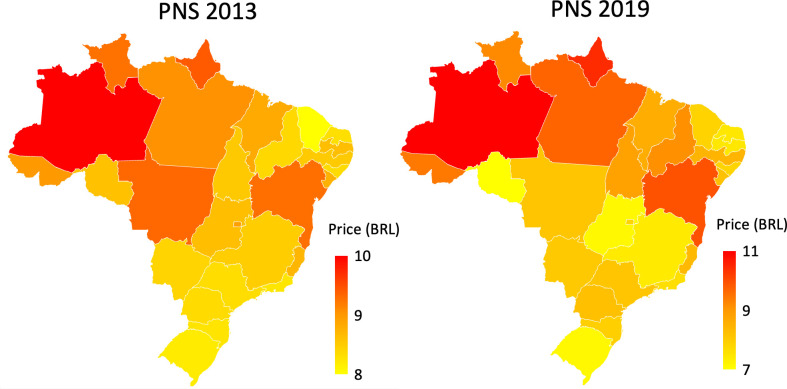
Average prices per 20-cigarette pack in the legal market across Brazilian states. Constant values of 2019. Source: National Health Survey (PNS) 2013 and PNS 2019. BRL, Brazilian real.

Since the good is highly similar but prices among different brands vary quite substantially, consumers may adjust to price changes by switching to a cheaper brand. To deal with a potential endogeneity bias and the problem of misreporting the price of the individuals’ last purchase, leading to measurement error and the well-known attenuation bias in the coefficients towards zero, reported unit prices are substituted with average prices within each state. Prices differ across states because part of the tobacco tax burden is given by a state-specific tax (ICMS (Imposto sobre Circulação de Mercadorias e Serviços)) that varies across states. In addition, logistic, distribution and transportation costs to the countryside are quite high due to poor infrastructure. Figure 1 reports average prices of legal cigarettes across the Brazilian states. Prices tend to be lower in producer states and higher in the countryside and in states with larger tax burden. Price dispersion increased from 2013 to 2019 probably due to a fixed value of BRL5.00 for the official minimum price since 2016, product differentiation and more brands entering the market.

The conditional price elasticities, $\varepsilon_{d}$ , are estimated from the baseline specification:

(1) $$\ln Q_{idst}=\alpha +\sum_{d}\varepsilon_{d}\ln P_{idst}I_{dt}+\beta X_{idst}+D_{s}+D_{t}+e_{idst}$$

where *Q_idst_* is the number of cigarettes smoked per day by individual *i* in income quartile *d,* federal state *s* and year *t*; *P_idst_* is the price that individual *i* actually paid per cigarette; *I_dt_* is a binary variable that indicates to which income quartile (or age cohort) the individual belongs; and the vector *X_idst_* includes control variables for age, education, years of smoking, income and gender. *D_s_* represents the state fixed effects, *D_t_* accounts for year fixed effects and *e_idst_* is the compound random error term of the regression.

To estimate price elasticities for the licit and illicit cigarette markets, we include a binary variable for these markets and interact it with all explanatory variables, as reported in equation (1′):

(1′) $$\ln Q_{idst}=\begin{array}{l} \alpha +\sum_{d}\varepsilon_{d}\ln P_{idst}I_{dt}+\sum_{d}\xi_{d}\ln P_{idst}I_{dt}I_{imt}+\beta X_{idst}+\\ \delta X_{idst}I_{imt}+D_{s}+D_{t}+D_{s}I_{imt}+D_{t}I_{imt}+e_{idst} \end{array}$$

where $I_{imt}$ is a dummy variable that assumes value of 1 for cigarettes purchased by individual *i* in the licit market in time *t* and 0 for purchases from the illicit market. As explained above, to account for potential endogeneity, unit prices are replaced by state average prices, in which case the variable $P_{idst}$ becomes $P_{dst}$ because there is price variation only across Brazilian states in this case. The interaction dummy is also adjusted accordingly to capture the average price of licit cigarettes across the states in the joint estimation of the price elasticities.

To calculate how tax adjustments affect cigarette consumption, one needs to be aware that a price change may induce individuals either to start smoking or to quit. This issue is also well known in the literature on tobacco, although, mainly due to data availability, not all studies are able to deal with the problem. Following the exposition in WHO,^19^ this study estimates the unconditional price elasticity related to the number of smokers from the following probit model.

(2) $$\mathrm{Pr}\left(S_{ist}\right)=\phi \left(\alpha +\gamma P_{st}+\delta X_{ist}+D_{r}+D_{t}\right)$$

The dependent variable in equation (2) is an indicator of whether individual *i* is a smoker or not, and *P_st_* is the average price in federal state *s* and year *t* of the individual reported price. The explanatory variables *X* are the same as in the conditional price elasticity estimation by equation (1), except that we use the state average cigarette prices without interaction terms, because once the coefficients are estimated, the non-linear probit model allows us to calculate the price elasticities for different income or age levels. For this reason, we also use region fixed effects (D_r_) instead of the more disaggregated federal state fixed effects. The price elasticity from the smoking decision model for an individual with average characteristics $\bar{X}=\frac{1}{N}\sum {\;}_{i=1}^{N}X_{i}$ is calculated as follows:

(3) $$\varepsilon_{u}\equiv \frac{\partial E(S|\bar{X})}{\partial P_{st}}\frac{P_{st}}{E(S|\bar{X})}$$

where ‘E(.)’ represents the expected value of the expression in parenthesis. It is important to note that this elasticity indicates the per cent change in smoking prevalence due to a per cent change in the cigarette prices.

## Results

### Conditional price elasticity of cigarette consumption

The estimations of the conditional price elasticity of cigarette consumption reported in table 2 show that the differences between the licit and illicit markets are substantial. Consumers in the illicit market are less sensitive to price changes.

**Table 2:** Conditional price elasticities by income quartile and market type

Market	(1)	(2a)	(2b)	(3)	(4a)	(4b)
	Both	Legal	Illegal	Both	Legal	Illegal
Price measure	Individual			State average		
1st quartile	−0.23***	−0.39***	−0.27***	−0.41***	−0.90***	−0.31***
	(0.02)	(0.04)	(0.04)	(0.07)	(0.17)	(0.10)
2nd quartile	−0.21***	−0.37***	−0.26***	−0.42***	−0.90***	−0.35***
	(0.02)	(0.04)	(0.04)	(0.07)	(0.17)	(0.10)
3rd quartile	−0.23***	−0.42***	−0.25***	−0.39***	−0.87***	−0.32*
	(0.02)	(0.04)	(0.04)	(0.07)	(0.17)	(0.10)
4th quartile	−0.30***	−0.44***	−0.35***	−0.36***	−0.87***	−0.20**
	(0.03)	(0.05)	(0.05)	(0.07)	(0.17)	(0.10)
Observations	10 326	10 326		10 326	10 326	
R^2^	0.09	0.10		0.06	0.07	
F-quartiles	5.15***	1.39	5.08***	3.06**	1.85	4.62***
F-markets		2.84**			4.83***	

The table reports coefficients of price elasticities by income quartile according to the estimation of equation (1). The White-Huber heteroscedasticity robust SEs are in parenthesis. Columns (1) and (3) indicate purchases of both licit and illicit cigarettes, while columns (2a) and (4a) refer only to licit cigarettes and (2b) and (4b) remit only to licit cigarettes. Columns (1), (2a) and (2b) refer to individually reported prices while columns (3), (4a) and (4b) indicate state average prices. The last two rows report the F-statistics for testing if the estimated elasticities are equal. When both markets are considered separately in columns (2a), (2b), (4a) and (4b), the equation is estimated jointly with all variables being interacted with a market indicator variable. The estimations are based on pooled data from 2013 and 2019. All regressions include controls for gender, age group, education group, year and log income. Federal state fixed effects were only included in the estimations in columns (1), (2a) and (2b). Estimations in columns (3), (4a) and (4b) include region fixed effects instead.

*, ** and *** indicate statistical significance at the 10%, 5% and 1% levels, respectively.

Plausible explanations are that the smaller number of brands, the limited number of sales points and other characteristics of an illegal market limit the smokers’ choices and flexibility to adjust their consumption behaviour due to price changes. A recent report on illicit cigarette market found that in five representative cities of the country there were only a small handful of illicit brands (Drope *et al* 20). Additionally, the observations may stem from the fact that smokers of illegal cigarettes show a higher degree of addiction in line with Chaloupka^3^ and Becker and Murphy.^2^ The differences in price elasticities may also be due to other intrinsic characteristics of the illegal cigarette consumers. Online supplemental appendix table A.1 shows that smokers of illegal products consume more cigarettes and have been smoking for a longer time, in line with the previous literature in Brazil (Ribeiro and Pinto^16^). Note that switching between the legal and illegal cigarette markets in response to price changes is very limited, as recent evidence from Divino *et al* 21 indicates.

Based on the individually reported price of cigarettes, price elasticities in the illegal market vary between −0.25 and −0.35, whereas in the legal market they range from −0.37 to −0.44. Notice that the estimated elasticities by income quartile are not statistically different from each other within the legal market, but they are in the illegal market, as illustrated by the F-test for quartiles at the end of columns (2a) and (2b), respectively. They are also statistically different when considering the entire cigarette market in column (1).

Columns (3), (4a) and (4b) in table 2 report the results when individually reported cigarette prices are replaced by average price within each federal state. The estimation of the price elasticities now stems exclusively from variations between state average prices and income quartiles. Consequently, the elasticities unambiguously increase in magnitude. This change is expected because the regional prices eliminate the endogeneity bias caused by possible adjustments to cheaper brands under a price increase. Again, the price elasticities are statistically different between the two markets, legal and illegal, and they are different across income quartiles in the aggregated and the illegal market, as indicated by the F-statistics reported in the last row of table 2.

Table 3 provides price elasticities for five different age cohorts. Based on the previous findings, the distinction between licit and illicit markets is maintained. Regardless of individually reported price or state average price as a regressor, the five price elasticities are statistically different across markets and across the age groups in all of the six estimations, as illustrated by the calculated F-statistics in the last row of the table. As in table 2, the price-elasticities in the illicit market are lower than in the licit market while the endogeneity of individual prices downwards bias the estimates. Columns (3), (4a) and (4b) in table 3 report the results for the price elasticities by age cohort using the state average cigarette prices in the pooled sample, the licit and illicit markets, respectively. In all three cases, younger smokers seem to be more price sensitive. The estimates range from –0.99 to –0.85 in the licit market and from –0.38 to –0.27 in the illicit market in columns (4a) and (4b).

**Table 3:** Conditional price elasticities by age cohorts and market type

Market	(1)	(2a)	(2b)	(3)	(4a)	(4b)
	Both	Legal	Illegal	Both	Legal	Illegal
Price measure	Individual			State average		
Aged 15–29	−0.17***	−0.30***	−0.23***	−0.47***	−0.99***	−0.38***
	(0.02)	(0.05)	(0.04)	(0.07)	(0.17)	(0.10)
Aged 30–39	−0.24***	−0.40***	−0.28***	−0.41***	−0.92***	−0.30***
	(0.02)	(0.04)	(0.04)	(0.07)	(0.17)	(0.10)
Aged 40–49	−0.26***	−0.45***	−0.28***	−0.38***	−0.89***	−0.28***
	(0.02)	(0.04)	(0.04)	(0.07)	(0.17)	(0.10)
Aged 50–59	−0.28***	−0.50***	−0.28***	−0.35***	−0.85***	−0.27***
	(0.02)	(0.04)	(0.04)	(0.07)	(0.17)	(0.10)
Aged 60+	−0.25***	−0.48***	−0.24***	−0.37***	−0.86***	−0.34***
	(0.02)	(0.04)	(0.04)	(0.07)	(0.17)	(0.10)
Observations	10 362	10 362		10 362	10 362	
R^2^	0.08	0.09		0.06	0.06	
F-age groups	10.27***	13.78***	2.35*	20.49***	3.85***	20.44***
F-markets		7.09**			4.11***	

Columns (1) and (3) indicate purchases of both licit and illicit cigarettes, while columns (2a) and (4a) refer only to licit cigarettes and (2b) and (4b) remit only to licit cigarettes. Columns (1), (2a) and (2b) refer to individually reported prices while columns (3), (4a) and (4b) indicate state average prices. The last two rows report the F-statistics for testing if the estimated elasticities are equal. When both markets are considered separately in columns (2a), (2b), (4a) and (4b), the equation is estimated jointly with all variables being interacted with a market indicator variable. The estimations are based on pooled data from 2013 and 2019. All regressions include controls for gender, age group, education group, year and log income. Fixed effects for federal states were only included in the estimations in columns (1), (2a) and (2b). Estimations in columns (3), (4a) and (4b) include region fixed effects instead.

*, ** and *** indicate statistical significance at the 10%, 5% and 1% levels, respectively.

### Unconditional price elasticity of smoking probability

Table 4 reports the unconditional price elasticities derived from a single probit estimation according to equation (2). The observed pattern across age and income groups is in line with the conditional price elasticity estimates. That is, younger and wealthier individuals show a slightly higher, not statistically significant, propensity to quit smoking in response to a cigarette price increase. This finding is in accordance with the level of addiction for these groups observed in the literature and in online supplemental appendix table A.1. Yet, the differences between the estimates across groups are much less pronounced than the ones from table 3. That is, for most Brazilians, a 10% cigarette price increase should reduce the number of smoking individuals by about 5%.

**Table 4:** Unconditional price elasticities from the smoking probability model by subgroups

**Age cohorts**	**15–29**	**30–39**	**40–49**	**50–59**	**60+**
	−0.51***	−0.50***	−0.48***	−0.44***	−0.50***
	(0.06)	(0.06)	(0.06)	(0.06)	(0.06)
**Income quartiles**	**Q1**	**Q2**	**Q3**	**Q4**	
	−0.48***	−0.48***	−0.48***	−0.51***	
	(0.06)	(0.06)	(0.06)	(0.06)	

The table shows the estimated price elasticities and their White-Huber heteroscedasticity robust SEs in parenthesis. All elasticities are calculated from a single probit estimation according to equation (2), where the smoking indicator is regressed on the observed mean price of cigarettes in each federal state and year, and we control for year, gender, education group, region and log income. The estimations are based on the entire sample of smokers and non-smokers pooled for the years 2013 and 2019 that contains 122 947 observations.

*** indicates significance at the 1% level.

## Discussion

We used two detailed individual surveys—the PNS from 2013 and 2019 (IBGE^6^)—to estimate cigarette price elasticities by income quartile and age groups and to account for the effects of the illicit cigarette market in Brazil. The information regarding registered brands and the official minimum price for a 20-cigarette pack allowed us to identify those consumers engaged in the illicit consumption. That is, purchases below this floor price are considered illicit. According to the results, there is a significant difference in the price elasticity estimations from the licit and illicit cigarette markets. Specifically, the smokers are less sensitive to price changes when consuming cigarettes from the illicit market (about –0.3) compared with elasticities about –0.9 in the licit market.

In addition, to the best of our knowledge, this is the first study to estimate price elasticities of cigarette demand by income and age groups. We find that younger and poorer individuals seem to be more price sensitive; however, the differences across income quartiles are less pronounced as compared with the age groups. Tax increases would thus particularly induce young adults and poorer households to smoke less.

Another important implication of tax increases is that they should have a smaller effect, if any, on prices in the illicit market. Obviously, a tax does not directly affect illicit cigarette prices but previous evidence from Brazil that compares the evolution of prices in the licit and illicit cigarette markets points out that illegal cigarette prices increase when taxes are raised because there is a strong positive correlation between licit and illicit prices (Brazilian Health Ministry^22 23^). Therefore, even if we suppose that there is a correlation between the two prices, the lower price sensitivity of smokers in the illicit market suggests that they would be less affected by tax changes that eventually ended up raising both prices. However, smokers of illicit cigarettes are still demanding medical treatment for tobacco-related diseases and losing future years of productive life because of cigarette-related diseases. The existence of an illicit cigarette market prevents the government from collecting tobacco tax revenues and weakens the social reach of price-oriented antismoking public policies by blunting the effectiveness of the tax increases. Public policies that target cigarette smuggling should always be a priority because they reduce the illicit trade itself and they increase effectiveness and efficiency of other tobacco control policies.

A potential limitation regarding the identification of the illegal market is that we cannot account for high-price illegal cigarettes when using the official minimum price as a threshold to define illicit cigarettes in the 2013 data. Moreover, if an individual purchased both illegal and legal cigarettes at the same time, it is not possible to distinguish these products because we can only classify them by the mean price, leading us to consider them as either illegal or legal cigarettes. There might be some variability in general levels of smoking by state due to state-specific tobacco control measures that are captured by the state average prices leading to biased coefficients. A final drawback is that the Deaton’s average price methodology was designed for relatively small geographical units, while our dataset is aggregate for the Brazilian states. Due to the low number of smokers in the data and the representativity of the survey at the federal state level, further disaggregation is unfeasible.

## Data Availability

Data are available in a public, open access repository.
