# Supplementary material for: Effects of the illicit market on the price elasticity of cigarette consumption in Brazil
Source: Tob Control. 2023 Dec 1;33(Suppl 2):s122–7. doi: 10.1136/tc-2022-057787 (PMC11187375; doi:10.1136/tc-2022-057787)
Supplement: Supplementary data [file tc-2022-057787supp001.pdf]

Appendix

For the sake of completeness, Table A.1 summarizes the descriptive statistics for the main variables in the sample. The years of 2013 and 2019 are pooled but the sample is divided into the three dimensions that are used in price elasticity estimations. Columns (1) to (4) divide the sample according to income quartiles, columns (5) and (6) distinguish between the licit and illicit markets and, finally, columns (7) and (8) show the characteristics for smokers and non-smokers.

Table A.1: Summary statistics by subgroups – 2013 and 2019

	(1)	(2)	(3)	(4)	(5)	(6)	(7)	(8)
**Sample:**	**Inc. Q1**	**Inc. Q2**	**Inc. Q3**	**Inc. Q4**	**Licit**	**Illicit**	**Non-sm.**	**smokers**
% Smokers	0.09	0.12	0.11	0.09	1.00	1.00	0.00	1.00
	[0.29]	[0.32]	[0.31]	[0.29]	[0.00]	[0.00]	[0.00]	[0.00]
Years of smoking	27.81	23.15	25.89	28.01	25.75	28.28	8.84	26.61
	[17.04]	[15.71]	[16.04]	[15.29]	[16.19]	[16.31]	[18.12]	[16.28]
Cigarettes Per day	10.29	10.33	11.46	12.26	10.51	12.09	0.00	11.05
	[9.22]	[8.83]	[9.21]	[9.67]	[8.96]	[9.85]	[0.00]	[9.29]
Price per pack	6.45	6.87	6.95	7.98	8.44	4.17	0.00	6.99
	[3.62]	[3.61]	[3.16]	[2.81]	[2.84]	[2.42]	[0.00]	[3.38]
% Income Cigarettes	0.18	0.08	0.06	0.03	0.10	0.10	0.00	0.10
	[0.30]	[0.08]	[0.06]	[0.03]	[0.18]	[0.23]	[0.00]	[0.20]
Age	45.68	41.62	44.56	47.80	44.94	45.77	46.69	45.22
	[15.35]	[14.23]	[14.19]	[13.62]	[14.59]	[14.65]	[17.45]	[14.16]
Educ. [1]	0.27	0.17	0.13	0.05	0.13	0.25	0.16	0.17
	[0.44]	[0.37]	[0.34]	[0.23]	[0.33]	[0.43]	[0.37]	[0.37]
Educ. [2]	0.47	0.45	0.41	0.26	0.35	0.49	0.31	0.40
	[0.50]	[0.50]	[0.49]	[0.44]	[0.48]	[0.50]	[0.46]	[0.49]
Educ. [3]	0.10	0.16	0.16	0.15	0.16	0.09	0.14	0.14
	[0.29]	[0.37]	[0.37]	[0.36]	[0.37]	[0.29]	[0.35]	[0.34]
Educ. [4]	0.17	0.22	0.30	0.54	0.59	0.17	0.38	0.29
	[0.37]	[0.41]	[0.46]	[0.50]	[0.49]	[0.37]	[0.49]	[0.46]
% Male	0.50	0.61	0.65	0.67	0.59	0.60	0.46	0.59
	[0.50]	[0.49]	[0.48]	[0.47]	[0.49]	[0.49]	[0.50]	[0.49]
Income	712.7	1164.1	1791.1	5789.7	2634.4	1388.5	2317.2	2211.1
	[323.4]	[118.5]	[318.9]	[5310]	[3729]	[1440]	[4362.5]	[3199.3]
Obs.	4,157	1,841	3,245	2,814	8,021	4,036	110,426	12,057

Notes: The table shows average values and standard deviation (in brackets) for the main variables in different subsamples using the sample weights. Columns 1 to 4 refer to the four income quartiles. Columns 5 and 6 divide the subgroup of smokers into those that bought their cigarettes in the licit and illicit markets, respectively. Columns 7 and 8 divide the entire sample according to smokers and non-smokers, respectively. The four education categories indicate the share of individuals with 4 or less years, between 5 and 9 years, between 10 and 13 years, and 14 or more years of education, respectively.

Males are overrepresented in the three highest income quartiles. Women represent 62 percent of the smokers in the lowest income quartile. The table confirms that poorer individuals, and those from the lower educational groups are more likely to buy cigarettes from the illicit market.
